# STUDY PROTOCOL: EXPOSURE IN VIRTUAL REALITY FOR SOCIAL ANXIETY DISORDER - a randomized controlled superiority trial comparing cognitive behavioral therapy with virtual reality based exposure to cognitive behavioral therapy with in vivo exposure

**DOI:** 10.1186/s12888-020-2453-4

**Published:** 2020-01-30

**Authors:** Lars Clemmensen, Stéphane Bouchard, Johan Rasmussen, Trine Theresa Holmberg, Jakob Hyldig Nielsen, Jens Richardt Møllegaard Jepsen, Mia Beck Lichtenstein

**Affiliations:** 1 Center for Telepsychiatry, Mental Health Services in the Region of Southern, Copenhagen, Denmark; 2 Department of Psychoeducation and Psychology, University du Québec en Outaouais, Gatineau, Canada; 3 Center for Clinical Intervention and Neuropsychiatric Schizophrenia Research (CINS) and Center for Neuropsychiatric Schizophrenia Research (CNSR), Mental Health Centre Glostrup, University of Copenhagen, Glostrup, Denmark

**Keywords:** Social anxiety, Virtual reality, Cognitive behavioral therapy, Exposure

## Abstract

**Background:**

Social Anxiety Disorder (SAD) is characterized by an intense fear of negative judgement by others. Cognitive Behavioral Therapy (CBT) is recommended for treatment, but a substantial part of individuals with SAD either do not seek treatment or drop-out. CBT with Virtual Reality (VR)-based exposure has several advantages compared to traditional exposure methods, mainly due to increased control of situational elements. The aim of the current study is to develop a CBT program containing VR-based exposure. The intervention is targeted to adult patients suffering from SAD and treatment effect will be assessed by changes in SAD symptoms.

**Methods:**

This article describes the study protocol of a Randomized Controlled Trial with three arms: 1) CBT with VR exposure based on 360° videos 2) CBT with in vivo exposure and 3) VR relaxation therapy. There will be 30 participants in each arm with a crossover at the end of the treatment period during which the participants in the third group will be randomly re-allocated to one of the two former groups. The treatment program consists of 10 weekly individual sessions with a psychologist, and a six month follow-up consisting of a questionnaire. The primary outcome measure is reduction in SAD symptoms which will be assessed with the Social Interaction Anxiety Scale (SIAS).

**Discussion:**

There are currently no published studies on CBT with VR exposure based on 360° videos for SAD treatment. Furthermore, the current study will be the first Danish SAD treatment program that includes VR technology.

**Trial registration:**

clinicaltrials.gov (NCT03973541) June 3rd 2019.

## Background

Social Anxiety Disorder (SAD) is a common yet underreported mental disorder with an estimated lifetime prevalence of 3–13% [1–3], and low rates of spontaneous remission [4]. Patients with SAD experience intense and persistent fear during social interactions, where he or she might be negatively evaluated by others (e.g. public speaking, shopping). Symptoms include a high degree of bodily symptoms, such as sweating, trembling and increased heart rate, causing avoidance behavior when exposed to social situations (e.g. education, work) and significant functional impairment [5]. SAD is therefore highly debilitating, and the majority of individuals with social anxiety reports numerous problems with individual and social adjustments, as well as impairment in academic and professional functioning [6].

SAD often co-occurs with other psychiatric conditions such as additional mood disorders, substance use disorders, and is significantly associated with suicidal ideation [7, 8]. Despite its frequency and severity, only between one third and half of people with SAD seek treatment [5, 9]. This may be linked to the nature of the disorder itself, as people with SAD avoid healthcare services like they would any other social interaction [7]. Factors such as embarrassment associated with help-seeking and fear of what others might think have been found to prevent individuals with SAD from seeking treatment [7]. In addition to the disorder-specific issues, futher barriers exists in regards to accessing treatment such as the lack of skilled therapists, lack of evidence-based treatments and long waiting lists [10]..

The recommended psychological treatment for SAD is the exposure technique imbedded in Cognitive Behavioral Therapy (CBT) [2, 5]. CBT for SAD combines cognitive restructuring with exposure [11]. In exposure-based therapy, exposures to social situations are used to test and disprove the patient’s predictions about the danger in a particular situation thereby developing new, realistic mental representations associated with the feared stimuli [12, 13]. Patients with SAD often have excessively high standards for social performance, and a strong fear of ridicule [12, 14]. Thus, it can be particularly helpful to encourage patients to behave in ways that they would consider unacceptable. i.e. intentionally acts against their excessively rigid rules for social interaction while observing the consequences [12, 14].

Exposure has traditionally taken place as either.

- In vivo: directly facing the feared situation.
- Imaginary exposure: imagining the feared situation (can be facilitated through pictures or videos).

In vivo exposure is effective [14] but is costly, time-consuming and situational elements, such as the reaction of others, are difficult to control [15, 16]. Furthermore, many patients are rather unwilling to expose themselves to the real situation since it is considered too frightening [17], and there is a risk of encountering familiar people revealing that the person is in therapy.

Conversely, imaginary exposure may lack realism and intensity [18], and can be difficult for people who are unable to imagine vividly, it is also easy to avoid imagining their phobia-inducing situations, or for the patient to overwhelm themselves with images [17, 19]. Recently, researchers and clinicians have started to use Virtual Reality (VR) to overcome these difficulties [15, 16].

### Virtual reality- based exposure

VR is the use of computer and behavioral interfaces to simulate the behavior of 3D entities that interact in real time with each other and with a user immersed via sensorimotor channels [20]. It immerses the user in a computer generated or video-based virtual environment [21] . This enviroment can be created using either computer graphics or 360° 3D videos [21–23]. Studies on the effect of VR-based treatment for different types of phobias (e.g. agoraphobia and fear of flying) have revealed great potential [21], and a study showed that 76% of participants preferred VR-based exposure over in vivo exposure [24]. In addition to high levels of preference, VR-based exposure does have several advantages:

- Virtual exposure scenarios can be very similar to real life situations [25], and it is possible to control and regulate situational factors, such as degree of exposure for the patient and the reactions of other people in the scenario [26].
- Exposure scenarios can be presented to the patients, while still in the comfort and safety of a therapeutic room [25],
- The flexibility of VR allows the patient to experience situations that are worse and more exaggerated than those that are likely to be encountered in real life [17]. Thus it is possible to expose patients to others’ negative reactions, in a safe environment where they can learn that this is not dangerous, and that they can handle even the social ridicule they fear the most.
- Patients know that it is not real but their minds and bodies behave as if it is real. Hence people will more easily face difficult situations in VR than in real life and be able to engage in more adaptive behaviors [27].
- VR sessions often requires less time than in vivo sessions, and can be planned more flexibly and for less costs [28]

Seven meta-analyzes [9, 29–34] have been published on CBT with VR-based exposure for anxiety, and six of these include studies on SAD [9, 29–31, 33, 34]. All found superior effect of CBT with VR-based exposure compared to imaginary exposure, and similar effects when compared to in vivo exposure, with a recent study finding superior effect of CBT with VR-based exposure even when compared to in vivo [28]. Treatment effects have been shown to persist over a number of years [27], and a recent meta-analysis found persistent benefits of treatment after CBT with VR-based exposure comparableto face-to-face therapy [17]. Furthermore, a meta-analysis found that the CBT with VR-based exposure promoted benefits which carry over to real life, and leads to significant behavior change in real-life situations, [32] this analysis however, did not include SAD specific studies [32].

These meta-analyzes include only five [28, 35–38] randomized controlled trials (RCTs) on VR-based treatment for SAD which, however, all found significant improvements in SAD after VR-based treatment. Regarding the individual studies, one study comparing VR exposure with a waitlist group found a significant higher improvement in measures of anxiety in the VR exposure group [35]. Two studies found no significant difference between VR-based and in vivo exposure [36, 37], and Bouchard et al. (2017) [28] found VR exposure to be superior to in vivo exposure, whereas Kampmann et al. (2016) [38] found that in vivo exposure without CBT was more effective than VR-based exposure without CBT. However, as exposure was not performed in the context of CBT, direct comparison to the other studies is not possible.

The current evidence thus supports the clinical efficacy of CBT with VR-based exposure, and it is suggested that if the potential of VR is fully explored it might be more effective than in vivo [28]. However, the fact that these meta-analyzes include a total of only six [28, 35–39] (RCTs) and only four of these compare CBT with VR-based exposure to both an in vivo and a control group [28, 37–39]. The strength of the evidence base is also weakened by the use of small sample sizes (*n* < 30) [29] [32, 40] and the use of waiting lists patients as control groups [41], as waiting list control designs may overestimate intervention effects [42]. Patients assigned to a waiting list appear to improve less than would be expected for people who are concerned about their behavior and are taking steps to change. This contrasts with studies not employing waiting list designs in which control group patients tend to improve [43].

So far, CBT with VR-based exposure for SAD has been conducted by the use of computer-generated social environments. This method requires the computer to recreate (render) in real time all the virtual stimuli in order to adapt the user’s movements and interactions with the virtual stimuli. The advantage of real time 3D rendering is that the user can explore the virtual environment at will and at his her or her own pace. The disadvantages it that it takes computer power and time, and all stimuli has to be synthetic (digitized). Also, it is difficult and expensive to develop [44]. Using 360° videos avoids some limitations of real time rendering of 3D stimuli as it is more realistic, less expensive and requires minimal training, and developers can use real stimuli. All this contributes to making it easier to implement. However, the user cannot fully explore the environment (i.e., cannot go in directions that were not filmed in the video) or at his or her own pace (i.e., event will occur as already planned and filmed in the video). Video-based CBT with VR-based exposure has not yet been applied to SAD, but a study on social anxiety in the context of psychosis revealed positive results [45].

In 2017 Centre for Telepsychiatry did a pilot study (manuscript submitted) investigating the ability of 360° videos to trigger an anxiety response in patients with SAD. We developed and produced three videos in a shopping center and tested them on nine SAD patients and nine matched controls. Our main finding was that the videos were effective in producing anxiety in SAD patients while the control group did not report any anxiety. Furthermore, the participants reported high levels of presence. Presence is the person’s subjective sensation of being there in the Virtual Environment (VE) and appears to be key when inducing emotions such as anxiety through technology [46].

### Aims and hypotheses

The aims of the study that will use the clinical protocol described in the paper are to develop a complete program of CBT with VR exposure based on 360° videos for adult patients suffering from SAD, and to evaluate the treatment effect on SAD symptoms. We plan to compare a group receiving CBT with VR-based exposure immersed in an environment based on 360 videos stimuli to a group receiving CBT with in vivo exposure and a group receiving VR relaxation treatment. Having VR relaxation treatment as control group is methodologically superior to a comparison to a waiting list and controls for any placebo effect.

It is hypothesized that:

- CBT with VR-based exposure will significantly reduce symptoms of SAD
- CBT with VR-based exposure will be more effective than both CBT with in vivo exposure and VR relaxation therapy at the end of treatment
- An effect on symptom reduction will sustain at 6 months follow-up

## Methods

### Design

The study is designed as a three arm randomized controlled trial comparing a group receiving CBT with VR-based exposure, a group receiving CBT with in vivo exposure and a group receiving VR relaxation.

### Participants and recruitment

Patients have to meet the following criteria:

#### Inclusion criteria

- Able to comprehend the Danish language.
- ≥18 years.
- Fulfill the diagnostic criteria for SAD according to ICD-10 (F40.1) or ICD-11 (6B04).

#### Exclusion criteria

- Previously diagnosed with psychosis, Autism Spectrum Disorder or severe depression.
- Participating in other psychotherapeutic treatments during the study.
- Substance dependence disorder.
- Severe cyber-sickness (corresponding to motion sickness).
- If on medication, medication must be stable for 6 months prior to the study and during the study (i.e., no changes in type of medication or dosage).

Patients are recruited from the mental health services in the Region of Southern Denmark and from the Internet clinic at the Centre for Telepsychiatry (CTP). Patients are also recruited from the job centers in this Danish region. Case managers in these centers have in some cases experienced difficulties in getting people hired due to psychological problems and specifically due to what might be SAD. Most case managers have an educational background as social workers and have received basic knowledge on mental health as part of their education. They will be further trained by the research leader to identify job seekers who have difficulties getting a job due to problems that might have its cause in SAD. The case managers will hand out written information material to potential patients. Furthermore, we will advertise on the homepages for the CTP, for the Region of Southern Denmark, and at a national health website sundhed.dk

Participants will receive written information material and be asked to complete an online version of the Social Interaction Anxiety Scale (SIAS) [47]. If the SAD patient meets the inclusion criteria they are invited to a meeting conducted either at the CTP or through video call for oral information and diagnostic assessment. All potential participants will have their diagnoses discussed at weekly clinical conference. Those who meet the inclusion criteria will be offered participation and be asked to sign an informed consent statement.

Study data will be collected and managed using the Research Electronic Data Capture (REDCap) tools [48] hosted at the mental health services in the Region of Southern Denmark. REDCap is a secure, state-of-the-art web-based application designed to support data capture for research studies.

### Randomization and blinding

**Fig. 1:**
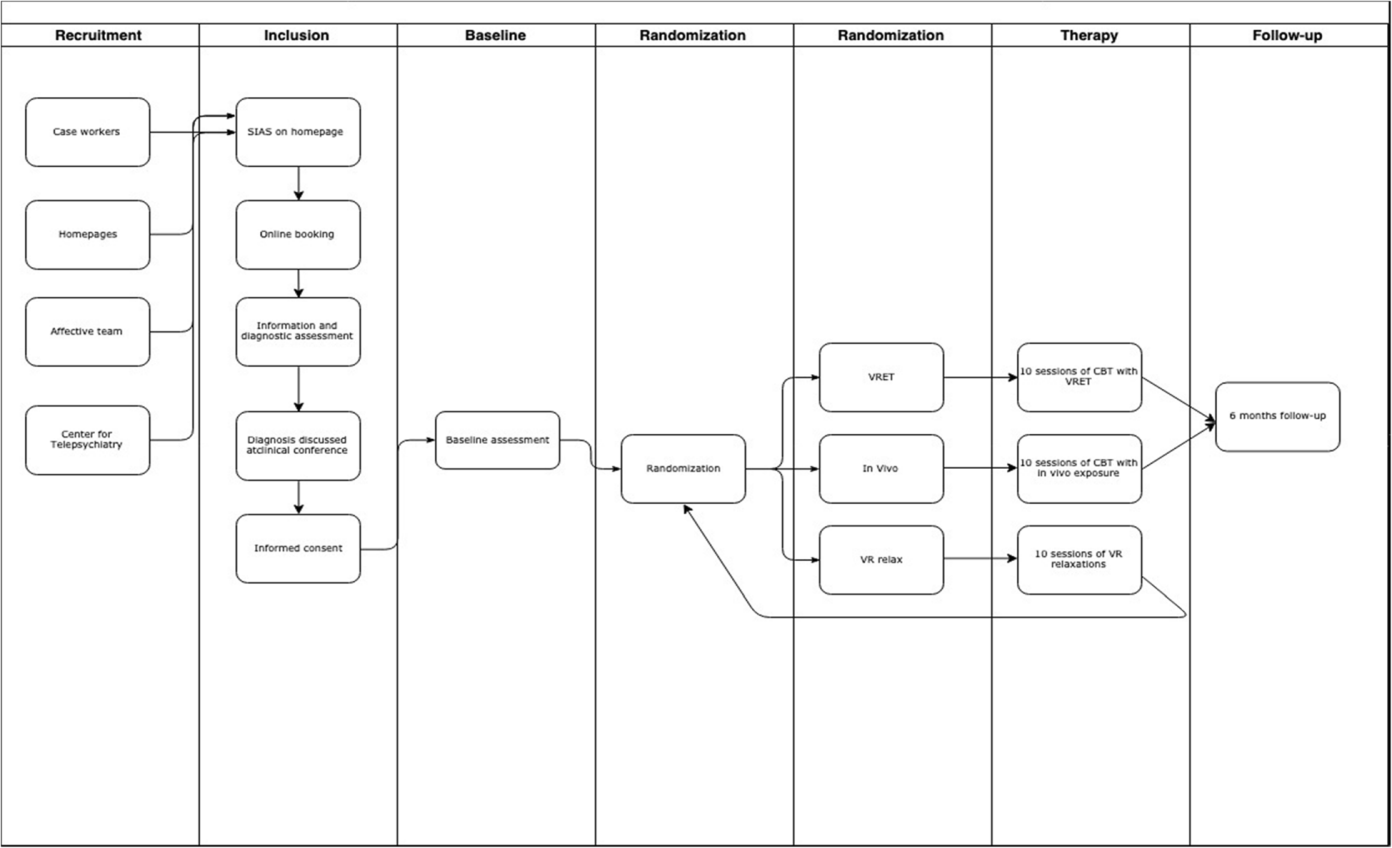
Overview of the patient pathways

Patients are randomly assigned (by gender, age and baseline severity of SAD) to one of the following three arms in ratio 1:1:1 via the computerized randomization function in REDCap: 1) Individual CBT with VR-based exposure in session 2) Individual CBT with in vivo exposure in session or 3) Individual VR relaxation therapy. Assignment will be kept blind for patients and clinicians until the first exposure session. An equal number of patients will be assigned to each group, but at the end of treatment patients in the third group will be randomly re-allocated to one of the two former groups. This reallocation is done to reduce the total number of patients needed to be recruited, and to avoid the ethical problem of not offering effective treatment. Six months after end of treatment the patients in group 1 and 2 will be re-assessed. There will not be a follow-up assessment of the patients in group 3, as they are re-allocated after week 10 (see Fig. 1).

### Sample size

Previous studies [28] report an approximately 10 point drop in SIAS for standard treatment with in vivo exposure. However, a 20 point drop has been observed for CBT with VR-based exposure [28] with standard deviations around 15 points in all treatment groups. We calculated effect sizes and found that 38 subjects are required in each group to detect if VR treatment is significantly better than standard treatment with in vivo exposure in a t-test with 80% power at the 0.05 significance level using a two-sided hypothesis. However, patients in the VR relaxation group continues in one of the exposure groups, so only 76 subjects need to be recruited. To compensate for a possible 10% drop-out rate we plan to recruit 90 patients.

### Interventions

A treatment manual based on Bouchard and colleagues’ adaptation [28] of the model and approach of Clark & Wells [11, 49] and Craskes Inhibitory Learning Approach [50, 51] will be applied for therapy. Treatment will be conducted by two clinical psychologists who will receive supervision every second week. Ten percent of the sessions will be audio recorded to ensure adherence to the manual. Recorded sessions will be chosen evenly across the different stages of treatment to ensure that all aspects of the treatment are represented when adherence is controlled. Treatment will be conducted for 10 consecutive weekly 60 min sessions. In the first two treatment conditions sessions are scheduled as follows: Session one: Building a therapeutic alliance and psycho-education about social anxiety and safety and avoidance behaviors. Session two to four: Cognitive restructuring. Session five to nine: Exposure in either in vivo or VR. Session 10: Relapse prevention. To ensure a fair comparison of the effect of VR and in vivo exposure, these treatment arms are identical except for exposure. All exposure happens in session, and whereas the exposure scenarioes in the CBT with VR-based exposure arm are limited to the scenarios mentioned below,there is a free choice of situation in the in vivo arm. This is to ensure a treatment as close to treatment as usual as possible in this arm. A recent meta-analysis found between-session interventions to be a significant moderator of attrition in both in vivo and VR-based exposure treatment [52]. Thus, to reduce dropout rate, and to adapt to treatment as usual at the mental health services in the region, homework is encouraged.

### Cognitive behavioral therapy with virtual reality based exposure

CBT with VR-based exposure will use 360° 3D videos with three scenarios: a) riding a bus, b) going to a school cafeteria and c) a job interview. These scenarios were chosen based on clinical experience and frequently reported difficult situations in the literature [28, 38]. The order of the scenarios is jointly decided by the patient and therapist. In the videos, the patients can make choices which determine the further course of the exposure scenario. For example, in the bus scenario, the system informing the passengers about the upcoming bus stop is out of order. Therefore, the patient has to ask the driver to tell when this is his or her stop to exit, when they reach the relevant stop. Depending on whether or not the patient decides to do so, the video will skip to one of two alternative directions of the scenario. During the VR exposures the therapist will also motivate the patient towards acting in ways they consider socially unacceptable or threatening (such as sneezing or putting a napkin on his/her head) to elicit fear of ridicule, and to help the patient learn to bend their rigid rules for social interaction.

### Cognitive behavioral therapy with in vivo exposure

In vivo exposure consists of therapist-guided exposure which takes place either inside or outside the therapist’s office with active modelling from the therapist in early sessions.. Similarly to the VR-based exposure during in vivo exposure the therapist will motivate the patient towards acting in ways they consider socially unacceptable or threatening.

### Virtual reality relaxation therapy

VR relaxation therapy will consist of a 360° 3D scenario of swimming with dolphins, created by the dolphin swim club (www.thedolphinswimclub.com). Swimming with real wild dolphins has been shown to have a positive effect on anxiety, although not specifically on SAD [53].

### Assessment

Diagnostic assessment will be conducted with the short version of the Present State Examination (PSE) supplemented with the anxiety section from the full version. PSE is a semi-structured interview, intended to provide an objective evaluation of present mental disorders. It contains 140 items, each scored on a 3-point or 4-point scale [54].

### Primary and secondary outcome measures

The primary outcome is degree of severity of SAD symptoms, which will be assessed at baseline, before exposure sessions, post-treatment and at the 6 months follow-up, with the Social Interaction Anxiety Scale (SIAS). SIAS is a measure of anxiety in social interactional situations and consist of 20 items scored 0–4. The total score range from 0 to 80 with a score higher than 43 indicating that SAD is probable [47]. The scale has been used and validated in international research, has good psychometric properties [47, 55], and focus on the interactional aspects of SAD, more so than on situations in which the person may be observed or scrutinized by others.

As a measure of general functioning at baseline, post-treatment and at the follow-up we use the EQ-5D-5 L. The EQ-5D-5 L is a standardized instrument for measuring generic health status [56, 57]. To study the practical and financial resources needed for exposure sessions, we include the questionnaire ‘Specific Work for Exposure Applied in Therapy’ (SWEAT). SWEAT measures cost and efforts required to conduct exposure sessions, and is filled out by the therapist after each exposure session [58]. As a measure of the therapeutic alliance we use the Working Alliance Inventory-Short Revised (WAI-SR) questionnaire, which assesses three key aspects of the therapeutic alliance: (a) agreement on the tasks of therapy, (b) agreement on the goals of therapy and (c) development of an affective bond [59].

### Other measures

Symptoms of ADHD and severity of autism spectrum disorder (ASD) characteristics will be assessed at baseline with the adult ADHD self-report scale (ASRS v1.1) [60] and the Social Responsiveness Scale (SRS-2) [61] respectively. Both ADHD and ASD are highly comorbid with SAD, and both are characterized by frequent social difficulties [62, 63]. Although patients with a diagnosis of ADHD are excluded, subsyndromal symptoms might complicate treatment. Alcohol and drug use will be assessed with the Alcohol Use Disorders Identification Test (AUDIT) [64] and the drug use disorders identification test (DUDIT) [65] respectively. Depressive symptoms will be assessed with the Beck Depression Inventory-II (BDI-II) [66], which is the most widely used instrument for detecting depression. (see Table 1).

**Table 1:** Overview of measurements

	Instrument	Items	Screening baseline	Before exposure sessions	Weekly at exposure sessions	Post-treament	Follow-up
SAD	SIAS	20	+	+	–	+	+
Diagnoses	PSE	140	+	–	–	–	–
Cost and efforts required to conduct exposure sessions.	SWEAT	11	–	+	+	–	–
ASD characteristics	SRS-2	65	+	–	–	–	–
ADHD	ASRS	18	+	–	–	–	–
General functioning	EQ-5D-5 L	26	+	+	–	+	+
Working Alliance Inventory	WAI-SR	12	**–**	–	**–**	**+**	**–**
Alcohol use	AUDIT	10	**+**	+	**–**	**+**	**–**
Substance use	DUDIT	11	**+**	**+**	**–**	+	**–**
Depression	BDI-II	21	**+**	+	**–**	**+**	**–**

### Statistical methods

The effect of the treatment will be analyzed using a linear mixed effects (LME) model with the SIAS score as the outcome and treatment group and assessment point (baseline, before exposure sessions, post-treatment and six months follow-up) as the independent variables, allowing intercepts to vary between patients. The moderating effect of ADHD symptoms severity, autism spectrum characteristics, depressive symptoms, alcohol and drug use on the treatment outcome will be investigated by including them as covariates in the regression. Comparisons for the SWEAT will be performed with t-tests, set two-tailed.

### Institution

The study will be hosted at the research unit at the CTP in the Mental Health Services of Southern Denmark. The research unit operates in the broad and interdisciplinary research field called telepsychiatry and e-mental health. It has a strong emphasis on translational and applied clinical research of novel use of technology in the field of mental health. CTP has its own multidisciplinary team for development and implementation with competencies within agile and user driven development and management of innovative E-mental health solutions. The team collaborates closely with the research unit in order to support careful handling of tech-development throughout the process of development, research and clinical implementation. The location has been approved by the National Committee on Health Research Ethics for the Region of Southern Denmark (Project-ID S-20180085).

### Production of videos

The 360° 3D video scenarios will be produced by KHORA Virtual Reality Denmark. KHORA will plan and manage the whole development process and will be responsible for filming and editing the VR content and building the VR platform, and responsible for development of content design and testing protocol in relation to the technical quality. The development will be informed using input from researchers, clinicians and engineers throughout the process. Scenarios will be produced one at the time and pilot testing will be conducted before the next scenario is produced. Patients admitted at the Mental Health Services of Southern Denmark, and former psychiatric patients attending Værekstedet Maskine Amager (MMA) will be helping with the pilot testing. For the VR relaxation therapy scenarios from The Dolphin Swim Club which will be integrated in the app alongside the exposure scenarios.

## Discussion

There are currently no published RCT studies on CBT with VR exposure based on 360° videos as SAD treatment. Furthermore, the current study will be the first Danish SAD treatment program that includes VR technology in a manner that takes advantage of the therapeutic possibilities this technology provides. Positive findings will support the use of CBT with VR-based exposure as an alternative, or supplement, to in vivo exposure. This is important because we still need more effective treatment options for SAD, considering the early onset of the disorder [67] and the negative effects on social skills. If the treatment proves successful, it is the intention to introduce it as an alternative at the Centre for Telepsychiatry (CTP) and, in the longer term, other psychiatric departments. With time and further technical refinement, this therapy might be carried out in the home of the patients, and thereby be helpful for patients who would not otherwise seek treatment because of severe fear of social interactions.

### Timetable and research plan

The current study will be carried out during 2019–2022. We are currently developing, producing and piloting the videos, and plan to begin recruitment and treatment of patients at the end of 2019. During this period a manuscript for a study protocol article will also be prepared. Recruitment and treatment will continue throughout 2020. During the first half of 2021 the follow-up assessments will be carried out. Afterwards, data analysis will be conducted and manuscripts for three further publications in international journals will be prepared.

### Compensation

The study is covered by The Patient Compensation Association. In order to increase motivation and acknowledge efforts to answer questionnaires for research use, the patients are rewarded with a gift card worth 500 DKK. Following finalization of treatment in accordance with guidelines from the Danish Committee on Health Research Ethics. Furthermore patients will be offered transport compensation.

## Data Availability

Not applicable.

## Abbreviations

ADHD: Attention-Deficit/Hyperactivity Disorder
ASD: Autism Spectrum Disorder
ASRS: ADHD self-report scale
AUDIT: Alcohol Use Disorders Identification Test
BDI-II: Beck Depression Inventory-II
CBT with VR-based exposure: Cognitive Behavioral Therapy with Virtual Reality based Exposure
CBT: Cognitive Behavioral Therapy
CTP: Centre for Telepsychiatry
DUDIT: Drug Use Disorders Identification Test
PSE: Present State Examination
RCT: Randomized Controlled Trial
REDCap: Research Electronic Data Capture
SAD: Social Anxiety Disorder
SIAS: Social Interaction Anxiety Scale
SRS-2: Social Responsiveness Scale 2ed
SWEAT: Specific Work for Exposure Applied in Therapy
VR: Virtual Reality
WAI-SR: Working Alliance Inventory-Short Revised
